# Health Sciences—Evidence Based Practice Questionnaire (HS-EBP): Normative Data and Differential Profiles in Spanish Osteopathic Professionals

**DOI:** 10.3390/ijerph17228454

**Published:** 2020-11-15

**Authors:** Juan Carlos Fernández-Domínguez, Isabel Escobio-Prieto, Albert Sesé-Abad, Rafael Jiménez-López, Natalia Romero-Franco, Ángel Oliva-Pascual-Vaca

**Affiliations:** 1 Balearic Islands Health Research Institute (IdISBa), 07120 Palma, Spain; jcarlos.fernandez@uib.es (J.C.F.-D.); albert.sese@uib.es (A.S.-A.); rafa.jimenez@uib.es (R.J.-L.); 2 Department of Nursing and Physiotherapy, University of the Balearic Islands, 07122 Palma, Spain; narf52@gmail.com; 3 Department of Physiotherapy, Faculty of Nursing, Physiotherapy and Podiatry, Universidad de Sevilla, 41009 Sevilla, Spain; angeloliva@us.es; 4 Department of Psychology, University of the Balearic Islands, 07122 Palma, Spain; 5 Escuela de Osteopatía de Madrid, 28002 Madrid, Spain

**Keywords:** clustering procedure, evidence-based practice, Health-Sciences Evidence-Based Practice questionnaire, normative data, osteopathy, professional profiles

## Abstract

The main goal of this study was to obtain normative data of the scores of the Health-Sciences Evidence Based Practice (HS-EBP) questionnaire, and to analyse evidence-based practice (EBP) among potential clusters of osteopathy professionals in Spain. An online descriptive cross-sectional study has been applied. A total number of 443 Spanish practicing osteopaths answered a survey including the 5 dimensions of the HS-EBP questionnaire and sociodemographic, training, and practice variables using the “LimeSurvey” online platform. Results point out that the median scores for each five HS-EBP questionnaire dimensions were 95.00, 86.00, 78.00, 84.00 and 62.00 considering that the range of possible scores in each of the dimensions was: from 12 to 120 in dimensions 1, 4 and 5; from 14–140 in dimension 2; and from 10–100 in dimension 3. A clustering algorithm extracted 6 different profiles across the five HS-EBP latent dimensions: low scores in all dimensions (cluster 1); low scores in all dimensions but with medium scores in dimension 1 (cluster 2); mixed pattern of scores, low in dimensions 2 and 5 and medium in the rest of the dimensions; medium scores in all dimensions (cluster 4); high scores in all dimensions and low scores in dimension 5 (cluster 5); and high scores in all dimensions (cluster 6). Significant relationship was found among the response patterns in the clusters and: academic degree level, EBP training and training level, and work time invested in healthcare activity, research and teaching activity. These results allow a description of the actual level of EBP and differential profiles of Osteopathy care practice in Spain. Knowledge of normative scores of the HS-EBP questionnaire and identification of different predictors of Spanish osteopaths’ EBP, e.g., academic degree, EBP training and training level, work time invested in healthcare activity, research, and teaching activity, and having a working relationship with an accredited educational centre, enable a comprehensive evaluation of the EBP of osteopathic professionals and can also be useful for developing and implementing formative intervention programs for improving EBP practice in osteopathic practice.

## 1. Introduction

Evidence-based practice (EBP) is increasingly important worldwide for the improvement of healthcare quality by Health Services [1,2]. Healthcare professions have been asked to embrace the principles of EBP, and the osteopathic profession is not an exception [3,4]. With this ongoing movement towards EBP, osteopaths are required to take a more reflective stance towards their practice and to integrate research evidence into their clinical reasoning [5,6,7,8]. However, there is little information about EBP among osteopaths. United Kingdom osteopaths were found to generally support and have positive attitudes about EBP, have moderate-level skills in EBP, and be infrequently engaged in EBP activities in spite of being interested in improving their skills in the field [9,10,11]. Regarding Australian osteopaths, they mostly agreed that evidence from research had a moderate to high impact upon their current osteopathy practice; they also considered that osteopathy research is useful to help patients understand the benefits of osteopathy, to help other health professionals to understand the role of osteopathy in health care, and to provide scientific evidence for what they do as osteopaths [12].

EBP assessment in allied health professions is generally carried out through self-reported instruments. These kinds of instrument are profusely reported in the scientific literature [13,14,15,16], including the specific area of complementary and alternative medicine (CAM) [17,18]. As evidenced in a systematic review by Veziari et al. [19], instruments include the questionnaire adapted from the Survey of Attitude and Use of Evidence-Based Practices (EBASE) [20,21,22,23] (initially developed by Leach & Gillham [24]), or OBSTACLES, which assesses barriers to implementing EBP in CAM professionals [25].

However, existing instruments for EBP measurement using a transdisciplinary approach are still scarce, despite the fact that this characteristic is considered important and very useful in some review studies carried out on the subject [26]. Attempting to fill this gap, a new EBP transdisciplinary questionnaire, the Health-Sciences Evidence-Based questionnaire (HS-EBP) was developed and validated [27]. This recent tool is based on a comprehensive analysis of the theoretical conceptualisation and operationalisation of the EBP construct. Its psychometric validation followed the most rigorous international standards for test development [28]. HS-EBP is useful for EBP individual assessment and for creating and evaluating formative specific interventions to improve EBP [27]. Given that HS-EBP was developed under Classical Test Theory (CTT), a score does not have an absolute value but is relative to normative data (from the reference population). This kind of test generally needs reference values to determine the status of EBP in the local practice environment with regards to other organisations, or to develop comparisons between different patterns of provider [29,30,31].

Based on the current state of knowledge of the EBP construct, one of the emerging issues is to establish reference values that will enable test users to compare EBP levels among different environments, organisations, and Health services [32]. In this sense, normative data are frequently obtained from a reference population [33,34], especially in the field of psychology [35,36]. However, criterion-referenced testing is not possible yet due to the lack of reference criteria for the values of the different EBP questionnaires. To our knowledge, normative data extraction and analysis have not been implemented with any EBP instrument, either uni-disciplinary [37,38,39] or transdisciplinary [40,41,42,43].

There is no information about EBP among Spanish osteopaths. A previous study aiming to describe the characteristics of Spanish osteopaths has shown that 75% of them had a prior academic degree in physiotherapy [44]. The aim of the study was to obtain normative data from the HS-EBP questionnaire, and to detect and discuss EBP potential clusters of Spanish osteopathy professionals, taking into account sociodemographic, training, and professional practice variables.

## 2. Materials and Methods

### 2.1. Participants

Practicing osteopaths in Spain were targeted. Non-probabilistic intentional sampling of volunteers was used. To obtain the sample, collaboration was requested from the Spanish Osteopaths Professional Registry (Registro de Osteópatas Fisioterapeutas de España—ROFE) and osteopaths were also invited during training/professional specialisation courses. Additional variables related to socio-demographics, training, and practice characteristics used to detect EBP differential profiles were sex, academic degree, EBP training level, practice setting, professional sector, type of professional activity development centre, work time invested in different types of activity in daily professional practice, years of professional practice, weekly working hours, and teaching centre. Academic degree was classified as Diploma/Bachelor’s/Graduate, Master’s, and Doctorate). EBP training level was operationalised as “Basic”, understood as having done an/some introductory course/s in EBP, bibliographic search in electronic databases or similar; “Intermediate” understood as, in addition to the above, also having done a/some introductory course/s in research methodology (asking a research question, critical reading of scientific articles, interpretation of statistical results, or similar); and “Advanced” adds having done a/some training course/s in research (statistics and handling computer programmes). Regarding practice setting, this was classified as “rural”, “urban with fewer than 50,000 inhabitants”, and “urban with equal or more than 50,000 inhabitants”. Professional sector considered three categories: “Public”, “Private”, and “Concerted/Mixed”. Type of professional activity development centre was categorised as “Specialised care”, “Primary/Community care”, “Socio-Health centre”, “School system”, “University academic staff”, and “Others”. Work time invested in different types of activity in daily professional practice was operationalized as the approximate percentage of time invested in “Healthcare activity”, “Research”, “Teaching”, “Management”, and “Others”. Finally, the protocol included a variable that asked if the workplace where the professional carried out his/her main activity was an accredited educational centre [27].

The final sample of Spanish osteopaths was composed of 443 professionals out of the 602 participants who voluntarily followed the link enabled to answer the survey (73.6% response rate). Mean age was 33.95 years (*SD* = 7.10), with 45.1% females, and mean period of professional activity was 7.39 years (*SD* = 5.33). Most worked in their own practice 69.7% (*n* = 281) or specialised care 15.4% (*n* = 62). The remainder worked in social centres 8.2% (*n* = 33), primary/home care, 4.2% (*n* = 17), university academic staff, 2% (*n* = 8), and in the school system, 0.5% (*n* = 2).

Furthermore, a huge proportion 84.6% (*n* = 369) worked in the private sector while only 9.2% (*n* = 40) worked in the public sector; 56.7% (*n* = 249) worked in an urban environment greater than 50,000 inhabitants, 31.4% (*n* = 138) in an urban environment with fewer than 50,000 inhabitants, and 11.8% (*n* = 52) in a rural environment. Regarding academic degree, 9% (*n* = 40) had a doctorate degree and 49.2% a Master’s degree (*n* = 218). In addition, 48.8% (*n* = 216) reported training in EBP, 27.3% (*n* = 59) of whom had a Basic training level, 41.2% (*n* = 89) an Intermediate training level, and 31.5% (*n* = 68) an Advanced training level. (Table 1).

### 2.2. Procedure

An online descriptive cross-sectional study was carried out using the survey tool “Limesurvey” (https://www.limesurvey.org/en/). Information about the study purpose, the project research team, and the link to access the questionnaire were included in the cover letter attached to the request for collaboration sent to the ROFE. The protocol was an open survey and therefore not password protected.

Once the participant had accessed the programme, the tool also included information concerning data handling (anonymity), informed consent, approval by the Ethics Committee, and contact with both the principal investigator of the project and the computer-services manager in charge of platform maintenance and collection of study data. Only one response option was allowed per item. Questionnaires with any unanswered item were eliminated. After fully completing the questionnaire, a message of gratitude was sent out.

### 2.3. Instrument

The survey included the HS-EBP questionnaire to measure EBP, along with additional sociodemographic, training, and practice variables. The current version of the HS-EBP questionnaire is made up of 60 items. A five latent model operationalised the following domains/dimensions based on previous research: D1 “Beliefs and attitudes” (12 items), D2 “Results from scientific research” (14 items), D3 “Development of professional practice” (10 items), D4 “Assessment of results” (12 items) and D5 “Barriers and Facilitators” (12 items) [27]. Items have a declarative format and a Likert scale (ranging from 1 to 10): the higher the score, the greater the agreement. In this way, range of possible scores in each dimension was: from 12 to 120 for D1, D4, and D5; from 14–140 for D2; and from 10–100 for D3.

The questionnaire showed adequate reliability evidence, with Cronbach’s alpha coefficients of 0.93, 0.96, 0.84, 0.94, and 0.91, for the 5 dimensions, respectively. Intraclass Correlation Coefficients (ICC) also showed moderate agreement in D1, D4, and D5, acceptable in D3, and considerable in D2 [45]. Evidence of both content and apparent validity was properly found [28]. Evidence of latent structure validity using confirmatory factor analysis (CFA) was obtained, with good overall and analytic fit (*χ*^2^/*df* = 2.89; *RMSEA* = 0.049, with CI 90% *RMSEA* = (0.047, 0.050); *SRMR* = 0.067; *CFI* = 0.99). Criterion validity evidence was also obtained considering “dispositional resistance to change”, “burnout”, and “quality of professional life” as criterial variables; and convergent validity evidence with respect to other EBP instrument (EBPQ) was confirmed [37]. Finally, proper evidence of decision validity using the “prior level of training of subjects in EBP” as discrimination variable was also obtained [27].

### 2.4. Statistical Analysis

The statistical package [46] SPSS 23 was used to perform the data analysis. A descriptive analysis of the sociodemographic, training and practice variables was performed using absolute and relative frequencies for the qualitative and ordinal variables. The mean, SD, and 95% CI of the mean were used for quantitative variables. A Kolmogorov-Smirnov test was applied for testing normality of the variables’ distribution. A descriptive analysis through central tendency, dispersion, and shape measurements was carried out on the HS-EBP dimensions’ scores. Standard scores (normative data) corresponding to the five dimensions were estimated by establishing the centiles of the entire distribution.

To obtain osteopaths’ EBP profiles, first a clustering procedure (sample segmentation) was applied using the K-means technique (partitional clustering technique) to discover distinctive groups (differential response patterns) through HS-EBP’s dimensions. K-means algorithm randomly divides the cases into k-clusters (number k specified by the user), then iteratively assigns each case to the nearest of the k-clusters (Euclidean distance was used, and k = 6), either by moving it or leaving it where it is. At each step, cluster centroids are recalculated before the next case is assigned; the process stops when there are no new case reassignments.

Secondly, to analyse the differential profile of the clusters, a bivariant analysis was conducted to explore the relationship between the new clustering variable (6 ordinal categories) and the other variables. Specifically, the Chi-square test was used on categorical variables, complementing the statistical significance (*p*) with the effect size provided by Cramer’s V coefficient (Φ_C_). Cramer’s V is a measure of association that shows the strength of association (removing the sample effect size) between two categorical variables, with values from 0 (null association) to 1 (strongest association). When quantitative variables were analysed, the ANOVA test was used, with the adjusted R^2^ effect size (population estimation), multiple comparisons with Bonferroni adjustment when the ANOVA test was significant (*p* < 0.05), and a linear trend contrast.

### 2.5. Ethical Considerations

The study was approved by the Research Ethics Committee (REC) of the University of the Balearic Islands (registration number 3566), conducted according to the ethical guidelines of the Declaration of Helsinki, and data privacy was respected (in compliance with the provisions set out in Regulation (EU) 2016/679 of the European Parliament and of the council of 27 April 2016 on the protection of natural persons with regard to the processing of personal data and the free movement of such data) [47]. Consent for participation was assumed when participants submitted their completed questionnaire online.

## 3. Results

### 3.1. Normative Data

The scores for each HS-EBP dimension can be observed in Table 2. For D1 (Beliefs and Attitudes) a median of 95 points was obtained, with values for 1 and 3 quartiles of 83 and 105 points respectively (Inter Quartile Range, *IQR* = 22); while the mean was 93.18 points (SD = 17.07), with a 95% CI: 94.77–91.59. For D2 (Results from scientific research), the median score was 86 points, with values for 1 and 3 quartiles of 62 and 107 points respectively (*IQR* = 45), while the mean reached 76.47 points (*SD* = 12.25), with a 95% CI: 77.61–75.33. For D3 (Development of professional practice), a median of 78 points was obtained, with values for quartiles 1 and 3 of 69 and 85 points respectively (*IQR* = 16), while the mean was 84.40 points (*SD* = 29.07), with a 95% CI: 87.11–81.69. For D4 (Assessment of results), the median score was 84 points, with values for 1 and 3 quartiles of 69 and 97 points respectively (*IQR* = 28), while the mean score was 82.09 points (*SD* = 20.38), with a 95% CI: 83.99–80.19. Finally, for D5 (Barriers/Facilitators) a median of 62 points was obtained, with values for 1 and 3 quartiles of 41 and 82 points respectively (*IQR* = 41), while the mean was 62.58 points (*SD* = 25.06), with a 95% CI of 64.54–60.62. Kolmogorov-Smirnov normality test was statistically significant in all HS-EBP dimensions (60 items) (*p* ≤ 0.001). (Table 2).

Normative data using percentiles for the five dimensions of the HS-EBP questionnaire, with the sample of Spanish osteopaths as reference population, are shown in Table 3. 

### 3.2. Clusters and Differential Profiles

Based on the scores obtained for the HS-EBP dimensions, six different groups were obtained (Table 4): low scores in all dimensions (cluster 1); low scores in all dimensions but with medium scores in D1 (cluster 2); mixed pattern of scores, low (in D2 and D5) and medium in the rest of the dimensions (D1, D3 and D4) (cluster 3); medium scores in all dimensions (cluster 4); high scores in all dimensions and low scores in D5 (cluster 5); and high scores in all dimensions (cluster 6).

Comparing the clusters with sociodemographic, training, and professional practice, a statistically significant relationships were found by sex (*p* < 0.001; Φc = 0.232), degree (*p* < 0.001; Φc = 0.200), EBP training and EBP training level (*p* < 0.001; Φc = 0.284 and *p* < 0.001; Φc = 0.273, respectively), practice setting (*p* = 0.003; Φc = 0.173), and teaching centre (*p* < 0.001; Φc = 0.236). In this way, a higher percentage of men (71.6%) was observed in the cluster with a high score in the five dimensions (cluster 6), while in the rest of the clusters this percentage decreased significantly, with a tendency to equalize with the subpopulation of women. Regarding having an academic degree, a higher percentage of doctorates (21.1%) was seen in the cluster with a high score (cluster 6), while in the remaining clusters this percentage also decreased significantly. The percentage of master’s students showed less variability between the different clusters, and without the clear trend observed in doctorates. Lastly, in the case of Diploma/Bachelor’s/Graduate professionals, an inverse percentage pattern was observed to that found among doctorate graduates (50% of professionals with low scores belonged to this group). Meanwhile, there was a higher percentage of those trained in EBP (71.3%) in the cluster with a high score (cluster 6), and also of those with an Intermediate (35.1%) and Advanced (51.9%) level of training in EBP.

For the remaining the clusters, both the percentage of those trained in EBP and those with an Advanced level decreased significantly. Furthermore, a higher percentage of professionals who carried out their work in an urban area with more than 50,000 inhabitants was seen in the cluster with a high score (cluster 6) (65.7%), while in the remaining clusters this percentage decreased significantly. Conversely, within the subpopulation of professionals who practiced in rural areas, the cluster with a low score (cluster 1) was the one with the highest quota of representation (33.3%). Lastly, a higher percentage of participants linked to teaching centres could be seen in the cluster with a high score (cluster 6) (37%) than in the cluster with a low score, where 86.1% were not linked to accredited educational centres.

A statistically significant relationship was also obtained between work time invested in healthcare activity, research, and teaching activity (*F* = 10.79, *df* = 441, *p* < 0.001, *adjusted R*^2^ = 9.6%; *F* = 16.23, *df* = 441, *p* < 0.001, *adjusted R*^2^ = 15.1%; and *F* = 6.05, *df* = 442, *p* < 0.001, *adjusted R*^2^ = 5.4%; respectively), as well as in the years of professional practice (*F* = 8.64, df = 440, *p* < 0.001, *adjusted R*^2^ = 7.6%) and response patterns (clusters) (Table 5).

Regarding the percentage of time devoted to the activity of care, research, and teaching, as well as years of professional practice, significant differences were found exclusively between the cluster with a high score (cluster 6) and the remaining clusters. There were no significant differences among the other clusters analysed considering these variables. Nevertheless, there was a statistically significant trend in all these variables, such that the higher the percentage of time devoted to research and teaching, and the lower the percentage of time devoted to care (*p* < 0.001 in all cases), as well as the more years of activity (*p* = 0.003), the higher the score in all groups.

Finally, in relation to the time (hours) of dedication to weekly work, there was also a significant relationship between weekly hours of dedication and the grouping variable (response patterns) (*F* = 6.61, *df* = 439, *p* < 0.001, *adjusted R*^2^ = 5.9%). The linear trend contrast was also significant (*p* = 0.006), even though in this case no clear growth pattern was observed, although this was found with years of activity.

**Figure 1:**
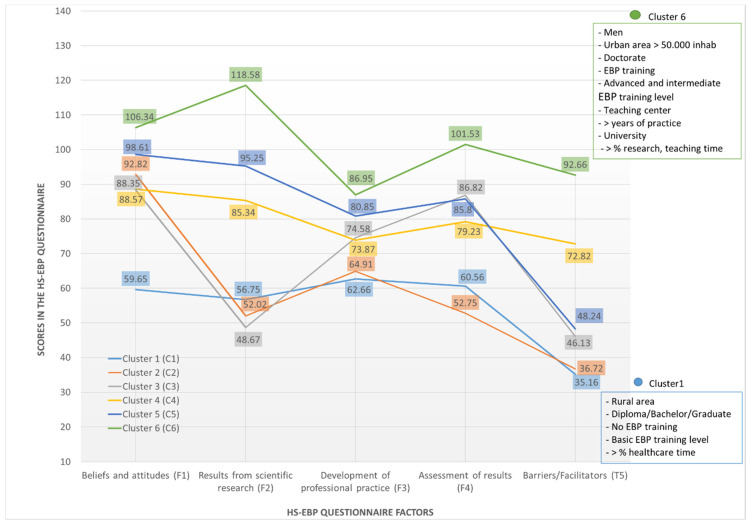
Line diagram showing clusters’ scores behavior through the five HS-EBP dimensions.

Conversely, there was no significant relationship among the response patterns (clusters) across the five dimensions analysed and the type of centre where the professional activity was carried out (*p* = 0.312), professional sector (*p* = 0.479), or amount of work time invested in management activities or other activities (*p* = 0.465 and *p* = 0.446, respectively). Figure 1 represents the EBP profile of each of the clusters according to the scores obtained for each of HS-EBP’s five dimensions. In addition, those variables on which significant profile differences are seen for the low (C1) and high (C6) clusters are described.

## 4. Discussion

Development of interventions and strategies oriented towards the implementation of EBP principles in clinical practice and to the improvement of healthcare system management, the main focus of future research in the area of osteopathy, can be facilitated by the availability of normative scoring data and the knowledge of sociodemographic, training, and practice characteristics for which significant differences in the use of EBP have been observed [9].

Firstly, it should be mentioned that, although osteopathy is not currently an independently regulated healthcare profession in Spain, the sociodemographic profile of the osteopathic professionals in our study is quite similar to that obtained in other studies carried out in other countries [48,49,50], although with a larger percentage of higher qualifications [12,48] (Master’s or doctorate) and greater dedication to research and higher education [10] in the sample of Spanish osteopaths.

Currently no studies have been conducted on normative data in relation to any other EBP measurement instrument. The normative scoring data obtained in the study enables the comparability of EBP between different environments of osteopathic practice in different organizations [51,52]. However, it must be taken into account that the results obtained are only a reflection of the current state of osteopathic EBP in Spain. In this sense, as indicated by Sundberg et al. [9]:

“future research should determine ideal levels of EBP activity for practicing osteopaths, and whether that might vary between different clinical settings and scopes of practice (e.g., Europe vs. US) … and further, whether different levels of EBP activity translate into poorer or improved patient outcomes” (p. 8).

It can be seen that in the dimensions related to the EBP process, D2, D3, and D4, the value of the 50th percentile is located in the middle of the range of possible scores in each of those dimensions. These results match with the results from other previous studies in which it is appreciated that research engagement and capacity relating to osteopathy practice remains limited [10,12]. Regarding D1 (beliefs and attitudes towards EBP), medium-high values are shown, similar to those obtained in other recent studies [9,10,11,12]. In the perception of organisational factors as barriers or facilitators for EBP implementation (D5), the values of the 50th percentile are only slightly above the average score of their possible score range. Therefore, it would seem that organisational factors are not considered by professionals as an essential element of EBP model implementation, similarly to what was reported in another study [10]. In some studies, it is shown that osteopaths make less use of scientific evidence in their clinical practice than other manual therapy professions do [20,23]. This could be explained, according to Leach et al. [24], by the different levels of research/EBP training, culture and opportunity for engagement in EBP in relation to the osteopathic profession [10]. Nevertheless, when interpreting the differences found between the different studies concerning EBP, it must be borne in mind that the nature and type of osteopathic care provided varies between countries in Europe and also internationally, and this, in addition to the use of different measurement instruments, makes it difficult to compare these variables [12,48,49].

In some studies, analyses of different variables that influence different “constructs” related to EBP (attitudes, knowledge, skills, use, and/or barriers/facilitators) have been carried out in the field of osteopathy; however, they have only been used to describe the characteristics of the sample participating in the study. To our knowledge, there are no studies on any published EBP measurement instrument in the field of osteopathy, or in the rest of complementary and alternative medicine (CAM), in which the factors influencing EBP have been analysed with the methodology used in this study; that is, evaluating these factors based on different levels (clusters) of evidence-based practitioner scores, despite the enormous interest that this could attract.

In the present study, six groups/clusters of EBP scores of osteopathic professionals were detected, enabling the determination of a series of differential profiles of EPB. Only two clusters, defined as cluster 1 (C1) and cluster 6 (C6), followed a uniform scoring pattern in all the dimensions analysed: high in the case of C6, and low in C1.

Knowledge of the sociodemographic, training, and practice profile of C6 subjects could be especially useful in allowing the detection of potential mentorship for EBP implementation processes in health organisations that consider the implementation of practices based on scientific evidence a priority objective [53]. According to the results of our study, this profile corresponds to male professionals (71.6%), residents in urban areas of more than 50,000 inhabitants (65.7%), with a doctor’s degree (21.1%), training in EBP (71.3%) especially at an Advanced level (51.9%), a greater number of years of working activity (9.91 years on average), a higher percentage of working time devoted to research and teaching (11.66% and 17.58%. respectively, on average in relation to total time), and a lower percentage of time devoted to healthcare activity/clinical practice (58.47% of total time).

These results match most of those obtained in previous studies, in which a significant positive association is found between use, skills, and attitudes towards EBP and working in urban areas [9,10], as well as a higher academic qualification level [9] and weekly hours devoted to both research and teaching [9]. However, there are some discrepancies concerning the results obtained in this study in relation to certain variables. Thus, in some studies, a negative, albeit weak, correlation is found between the use of EBP and the number of years of activity practicing osteopathy [9] or even in relation to attitudes towards it [10]. This divergence could be justified by the still recent development of training in osteopathy in Spain, which is reflected in the low average age of our population; i.e., even the oldest subjects in our sample had already received, on the whole, training in EBP during their academic training, as also seemed to occur in the study on physical therapists in the USA carried out by Jette et al. [54].

In the same way, an analysis of the profile of C1 would also allow the identification of professionals with high risk of behaviours far from the EBP paradigm, upon which to establish individualised diagnoses of their behaviour and/or competence in EBP through the use of appropriate measurement instruments, as well as to facilitate decision-making regarding the development and implementation of interventional measures, if deemed appropriate.

According to the results of our study, the profile of these subjects is almost opposite to that of C6, highlighting their lower training in PBE (only 50%), with a large percentage of their working time devoted to healthcare practice (76, 88%), and a somewhat more pronounced presence of female professionals (53.1%), without advanced academic training (50% graduates without postgraduate), and of those resident in rural areas (33.3%). The rest of the clusters do not follow such a uniform scoring pattern. In this sense, the most outstanding would be related to C2 and the comparison between C4 and C5.

C2 presents low scores in all dimensions, but with scores close to the global mean in D1; i.e., these would be professionals with low EBP behaviours and a perception of important barriers to its development, but with relatively good beliefs and attitudes towards it. The subjects belonging to this group, since they already show a good predisposition towards EBP, could be susceptible to interventions aimed at both improving knowledge and skills regarding EBP at an individual level, and at addressing the prevailing organisational barriers in each of their work environments. This cluster is characterised by a profile similar to that of C1, although with some notable differences in terms of degree qualifications, namely that the number of Master’s and doctorate graduates increases by 7.9% and 3.5%, with a significantly reduced average number of years of activity (5.36 years on average), a lower percentage of residents in rural areas (14.3%), and, unexpectedly, a decreased percentage of osteopaths trained in EBP (35.1%), as well as at the Intermediate and Advanced level of training. Cluster 3 (mixed), with a pattern of low scores (D2 and D5) and medium scores in the rest of the dimensions (D1, D3, and D4), would be made up of professionals with high beliefs in and attitudes towards EBP, but who show deficiencies, especially in terms of knowledge-skills in EBP and who seem to base their practice to a greater extent on non-regulated/informal sources of information. Subjects in C3 show notable barriers to the implementation of EBP, as in C1 and C2, although to a lesser extent.

Cluster 5 presents high scores in all dimensions, i.e., adequate attitudes to and behaviours regarding EBP, but with relatively low scores in D5, which seem inconsistent with respect to the scores obtained in that same dimension in the rest of the clusters. C4 presents medium scores in all dimensions, but with an inverse pattern to C5 in D5. This difference in the pattern of both clusters could be justified because C4 professionals had greater dedication to teaching (12.81% of total time vs. 10.58% in C5), which is consistent with a higher percentage of professionals linked to educational centres in C4 (30.9%). In C5 subjects, it would seem that interventions or strategies for the implementation of the EBP should be especially directed towards the analysis and specific approach of the organisational barriers that are causing a greater perception of obstacles to the implementation of EBP.

The main limitation of this study comes from the data collection system used (the online survey tool “Limesurvey”); the subjects who answered the questionnaire have skills in information technology, which can contribute to facilitate their access to scientific knowledge with respect to those professionals who do not possess this kind of skill. Further, as with any survey, especially in the case of the evaluation of attitudes, it is possible that participants with a great interest in EBP might be more likely to have participated in the study (self-selection bias). The social desirability bias inherent in this type of instrument would also contribute; this is reflected in the high values in the different questionnaire dimensions, especially in the items related to D1 (attitudes and beliefs).

## 5. Conclusions

Knowledge of normative scores from the HS-EBP questionnaire as well as identification of different predictors of EBP including sociodemographic, training, and practice features of Spanish osteopaths will enable a comprehensive evaluation of the EBP of osteopathic professionals and allow the use of targeted strategies and interventions/implementation plans in order to aid the still necessary and desired change in EBP in daily clinical practice.

In the present study, the variables that are significantly associated with the profile of an evidence-based practitioner are: academic degree, EBP training and EBP training level, work time invested in healthcare activity, research, and teaching activity, and having a working relationship with an accredited educational centre.

A relationship is also found with respect to other variables such as sex, practice setting, weekly work dedication, and years of professional practice. Nevertheless, no association was found with variables of a sociodemographic type such as age, or others linked to practice, such as professional sector, type of professional activity development centre, and work time invested in management activities. With regard to all these aforementioned variables, there are notable disputes between the different studies published as far as their association with EBP is concerned, thus requiring further studies to be conducted on this issue.

## Figures and Tables

**Table 1:** Sample description.

Variables	Statistics
**Age**	*M* = 33.95 *SD* = 7.10
**Sex**	
Male	200 (45.10%)
Female	243 (54.90%)
**Years of professional activity** **Type of work**	*M* = 7.39 *SD* = 5.33
Own practice	281 (69.70%)
Specialised care	62 (15.40%)
Social centres	33 (8.20%)
Primary/home care	17 (4.20%)
University academic staff	8 (2.00%)
School system	2 (0.50%)
**Context**	
Private	369 (84.60%)
Public	40 (9.20%)
**Urban environment**	
≥50,000 inhabitants	249 (56.70%)
<50,000 inhabitants	138 (31.40%)
Rural	52 (11.80%)
**Academic degree**	
PhD	40 (9.00%)
Master	218 (49.20%)
Other	185 (41.80%)
**EBP Training**	
Yes	216 (48.80%)
Basic	59 (27.30%)
Intermediate	89 (41.20%)
Advanced	68 (31.50%)
No	227 (51.20%)

**Table 2:** Descriptive statistics and normality tests of Health-Sciences Evidence Based Practice (HS-EBP) dimensions.

	Beliefs and Attitudes (D1)	Results from Scientific Research (D2)	Development of Professional Practice (D3)	Assessment of Results (D4)	Barriers/Facilitators (D5)
Median (P50)	95.00	86.00	78.00	84.00	62.00
P25–P75	83.00–105.00	62.00–107.00	69.00–85.00	69.00–97.00	41.00–82.00
Range	12.00–120.00	14.00–140.00	10.00–100.00	12.00–120.00	12.00–120.00
Asim. (EE) CI 95%	−0.71 (0.08) [−0.87; −0.55]	−0.19 (0.08) [−0.35; −0.03]	−0.70 (0.08) [−0.86; −0.54]	−0.49 (0.08) [−0.65; −0.33]	0.10 (0.08) [−0.06; 0.26]
Kurtosis (EE) CI 95%	0.68 (0.16) [0.36; 1.00]	−0.75 (0.16) [−1.07; −0.43]	1.11 (0.16) [0.79; 1.43]	−0.16 (0.16) [−0.48; 0.16]	−0.92 (0.16) [−1.24; −0.60]
K-S test	*p* = 0.001	*p* = 0.043	*p* < 0.001	*p* < 0.001	*p* < 0.001

*Note:* K-S test = Kolmogorov-Smirnov test.

**Table 3:** Percentiles distribution of the HS-EBP five dimensions.

Percentiles	D1	D2	D3	D4	D5
1	39.44	21.40	36.76	27.00	15.00
5	63.20	34.40	56.00	46.00	25.00
10	70.40	44.00	60.40	53.40	30.00
15	76.00	51.00	63.00	60.00	34.00
20	79.00	56.00	67.00	65.00	37.80
25	83.00	62.00	69.00	69.00	41.00
30	86.00	68.00	72.00	73.00	45.00
35	88.00	73.40	73.00	75.00	49.00
40	91.00	79.00	75.00	78.00	54.60
45	92.80	83.00	76.00	82.00	58.00
50	95.00	86.00	78.00	84.00	62.00
55	97.00	90.00	79.00	87.00	66.00
60	99.00	94.00	80.00	90.00	70.40
65	102.00	97.60	82.00	92.00	74.60
70	103.00	103.00	83.00	94.00	79.00
75	105.00	107.00	85.00	97.00	82.00
80	108.00	111.20	87.00	100.00	86.00
85	111.00	115.40	89.00	102.00	91.00
90	114.60	122.00	91.00	107.60	97.00
95	120.00	131.00	94.80	113.00	105.00
99	120.00	139.56	100.00	120.00	115.00

**Table 4:** Description of Clusters across the HS-EBP latent structure.

		Beliefs and Attitudes (D1) Range: 12–120	Results from Scientific Research (D2) Range: 14–140	Professional Practice Development (D3) Range: 10–100	Assessment of Results (D4) Range: 12–120	Barriers/Facilitators (D5) Range: 12–120
CLUSTER 1	*Mean*	59.65	56.75	62.66	60.56	35.16
	SD *	13.88	17.55	13.08	16.45	11.80
	*N*	32	32	32	32	32
CLUSTER 2	*Mean*	92.82	52.02	64.91	52.75	36.72
	SD *	10.26	16.57	11.57	13.82	15.03
	*N*	57	57	57	57	57
CLUSTER 3	*Mean*	88.35	48.67	74.58	86.82	46.13
	SD *	13.40	13.51	9.95	12.12	14.97
	*N*	55	55	55	55	55
CLUSTER 4	*Mean*	88.57	85.34	73.87	79.23	72.82
	SD *	13.77	12.92	8.82	13.51	11.20
	*N*	110	110	110	110	110
CLUSTER 5	*Mean*	98.61	95.25	80.85	85.80	48.24
	SD *	13.65	13.39	8.33	12.80	12.48
	*N*	80	80	80	80	80
CLUSTER 6	*Mean*	106.34	118.58	86.95	101.53	92.66
	SD *	10.02	11.85	7.50	11.96	13.22
	*N*	109	109	109	109	109
TOTAL	*Mean*	93.18	84.40	76.47	82.09	62.58
	SD *	17.07	29.07	12.25	20.38	25.06
	*N*	443	443	443	443	443

* SD = Standard deviation.

**Table 5:** One-way ANOVA with post-hoc Bonferroni tests to compare clusters (profiles) with professional practice related variables.

		**Clusters (Profiles)**					
		**Cluster 1** **(Low) Mean *(SD)***	**Cluster 2** **Mean *(SD)***	**Cluster 3** **Mean *(SD)***	**Cluster 4** **Mean *(SD)***	**Cluster 5** **Mean *(SD)***	**Cluster 6** **(High) Mean *(SD)***
Work time invested in different types of professional activity (daily)	% healthcare	76.88 ^a^ (23.28)	75.07 ^b^ (25.23)	79.43 ^c^ (16.73)	71.38 ^d^ (20.03)	73.83 ^e^ (18.23)	58.47 ^abcde^ (22.15)
	% research	6.69 ^a^ (14.78)	3.28 ^b^ (6.34)	1.72 ^c^ (3.29)	5.66 ^d^ (7.45)	4.86 ^e^ (6.40)	11.66 ^abcde^ (8.60)
	% teaching	7.47 ^a^ (12.67)	9.63 ^b^ (21.23)	5.49 ^c^ (10.24)	12.81 (14.81)	10.58 ^d^ (13.89)	17.58 ^abcd^ (15.23)
Years of professional practice		7.62 (6.67)	5.36 ^a^ (3.96)	5.58 ^b^ (4.66)	7.25 ^c^ (4.66)	6.78 ^d^ (5.17)	9.91 ^abcd^ (5.71)
Weekly Working hours		24.71 (12.24)	19.41 ^ac^ (13.40)	25.00 (13.93)	29.33 ^ab^ (13.03)	23.54 ^bd^ (14.44)	29.93 ^cd^ (12.67)

*Note*: Within the same quantitative variable, the means values by clusters with the same superscript letter (a–e) are significantly different from a statistical point of view. In all cases the difference is significant with *p* < 0.05.
